# Single-Subject Grey Matter Graphs in Alzheimer's Disease

**DOI:** 10.1371/journal.pone.0058921

**Published:** 2013-03-11

**Authors:** Betty M. Tijms, Christiane Möller, Hugo Vrenken, Alle Meije Wink, Willem de Haan, Wiesje M. van der Flier, Cornelis J. Stam, Philip Scheltens, Frederik Barkhof

**Affiliations:** 1 Alzheimer Center and Department of Neurology, VU University Medical Center, Amsterdam, The Netherlands; 2 Department of Radiology, VU University Medical Center, Amsterdam, The Netherlands; 3 Department of Clinical Neurophysiology and MEG, VU University Medical Center, Amsterdam, The Netherlands; 4 Department of Epidemiology and Biostatistics, VU University Medical Center, Amsterdam, The Netherlands; Beijing Normal University, China

## Abstract

Coordinated patterns of cortical morphology have been described as structural graphs and previous research has demonstrated that properties of such graphs are altered in Alzheimer's disease (AD). However, it remains unknown how these alterations are related to cognitive deficits in individuals, as such graphs are restricted to group-level analysis. In the present study we investigated this question in *single-subject* grey matter networks. This new method extracts large-scale structural graphs where nodes represent small cortical regions that are connected by edges when they show statistical similarity. Using this method, unweighted and undirected networks were extracted from T1 weighted structural magnetic resonance imaging scans of 38 AD patients (19 female, average age 72±4 years) and 38 controls (19 females, average age 72±4 years). Group comparisons of standard graph properties were performed after correcting for grey matter volumetric measurements and were correlated to scores of general cognitive functioning. AD networks were characterised by a more random topology as indicated by a decreased small world coefficient (*p* = 3.53×10^−5^), decreased normalized clustering coefficient (*p* = 7.25×10^−6^) and decreased normalized path length (*p* = 1.91×10^−7^). Reduced normalized path length explained significantly (*p* = 0.004) more variance in measurements of general cognitive decline (32%) in comparison to volumetric measurements (9%). Altered path length of the parahippocampal gyrus, hippocampus, fusiform gyrus and precuneus showed the strongest relationship with cognitive decline. The present results suggest that single-subject grey matter graphs provide a concise quantification of cortical structure that has clinical value, which might be of particular importance for disease prognosis. These findings contribute to a better understanding of structural alterations and cognitive dysfunction in AD.

## Introduction

Alzheimer's disease (AD) is a progressive and disabling neurodegenerative disorder that accounts for approximately 50% to 80% of all dementia cases. AD is histopathologically defined by the presence of amyloid-β plaques and tau-related neurofibrillary tangles [1]–[7]. These plaques and tangles have been associated with local synaptic dysfunction, suggesting that AD is a dysconnectivity disease [4], [8]. In addition, specific patterns of cortical atrophy have been associated with AD, including memory related structures such as the hippocampus and other medial temporal lobe regions, and also the precuneus, cingulate and prefrontal areas (e.g., [1], [9]–[12]). However, clinical phenotypes often present with more complex cognitive deficits besides memory complaints and this might not be fully explained by atrophy patterns alone [13]. Precise associations between clinical phenotypes and different pathological processes are difficult to study, partly because local functional and structural disruptions can have unpredictable, widespread effects in complexly interconnected brain networks [14]. Graph theory provides tools to investigate the connectivity structure of such complex networks (i.e., graphs) that can be obtained with functional and also structural neuroimaging techniques [15]–[18].

Coordinated patterns of cortical morphology in structural magnetic resonance imaging (sMRI) scans have been described as graphs (e.g., [19]–[23]). The nodes in these structural graphs represent cortical areas that are considered to be connected when they covary in thickness or volume across subjects [24], [25], or when they show structural similarity within single-subjects [23]. Such graphs can be concisely quantified with graph theoretical properties. In agreement with other types of brain graphs (e.g., functional graphs that are derived from functional synchronisation patterns or anatomical graphs that are derived from diffusion tensor imaging, i.e., DTI) structural graphs have a non-trivial organisation of connectivity. Importantly, several properties of structural graphs are altered in AD [26]–[28]. Furthermore, these alterations are heterogeneously distributed across the brain, indicating that specific cortical areas contribute more to disease modifications of global network measurements. Structural graph disturbances have been interpreted to reflect decreased information processing efficiency, possibly mirroring functional disruptions in AD. However, the methodology of these previous grey matter graph studies restricted the investigation of graph properties to group-level analyses, and therefore the relationship between grey matter graph alterations and disease severity in individual patients still remains to be established.

Furthermore, while most functional studies have reported that graph topologies move towards more random connectivity configurations in AD [29]–[32], it is still unclear whether this also occurs in grey matter graphs. It has been proposed that the loss of highly interconnected areas renders graph topologies more random [29]. Moreover, highly interconnected areas might be specifically targeted by the disease, because these areas have been associated with increased amyloid-β deposition in AD [33], [34] and with increased vulnerability for activity-dependent degeneration [35]. Examining these topological alterations in single-subject grey matter graphs might provide more insight into the association of functional disruptions and coordinated changes in cortical morphology.

The present study addresses these questions by investigating graph properties of *single-subject* grey matter graphs for the first time in AD using a recently developed method [23]. Presently, we expected that if structural graphs are related to functional disruptions then AD structural graphs would be characterised by a more random topology than those from control subjects. We also expected that the contribution of local disruptions would be heterogeneously distributed across the cortex, with preferential involvement of highly interconnected areas. Furthermore, we hypothesised that within the AD group, a more random graph topology would be related to more severe cognitive decline.

## Methods

### Alzheimer patients and control subjects

In total 38 patients with probable Alzheimer's disease (AD) and 38 gender and age matched controls were recruited from the Alzheimer Centre of the VU University Medical Centre. All subjects received standard dementia screening that included a medical history, physical and neurological examination, cognitive examination, extensive neuropsychological evaluation, screening laboratory tests, an electroencephalogram (EEG), and a magnetic resonance imaging (MRI) scan. Patients were diagnosed during a multidisciplinary consensus meeting with probable AD when they fulfilled the criteria proposed by the National Institute on Aging and the Alzheimer's Association (NIA-AA) workgroup [36]. Subjects from the control group were people who visited the clinic with (mostly) memory complaints, but did not meet the criteria for MCI or major depression after standard dementia screening (as described above). General cognitive functioning was assessed using the mini-mental state examination (MMSE, [37]), which was part of the standard dementia screening. The Ethical Review Board of the VU University Medical Center Amsterdam approved the study, which was conducted in accordance with regional research regulations and conformed to the Declaration of Helsinki. All participants or their lawful caregivers provided written informed consent to use their clinical data for research purposes. Patients who declined to provide written informed consent were not disadvantaged in any other way by not participating in the study.

### Image acquisition and preprocessing

Neuroimaging of the subjects was carried out on a 3.0 Tesla scanner (SignaHDxt, GE Healthcare, Milwaukee, Wisconsin, USA) using a standard circularly polarised head coil with foam padding to restrict head motion. The scan protocol included a whole-brain 3D fast spoiled gradient-echo sequence (FSPGR) with a repetition time of 708 ms, an echo time of 7 ms, flip angle of 12°, 180 sagittal slices, field of view of 250 mm, slice thickness of 1 mm, and a voxel size of 0.98×0.98×1 mm^3^. All scans were reviewed for brain pathology other than atrophy by an experienced radiologist. The origin of the scans was automatically set to the anterior commissure using the linear transformation matrix to MNI space that was calculated in FSL-FLIRT [38]. Next, the structural T1 weighted images were segmented into cerebrospinal fluid, grey and white matter using Statistical Parametric Mapping software (SPM8; Functional Imaging Laboratory, University College London, London, UK) implemented in MATLAB 7.12 (MathWorks, Natick, MA). C.M. visually checked the quality of all segmentations.

### Single-subject grey matter networks

Single-subject grey matter graphs were based on intracortical similarity using a completely automated and data-driven method that has been previously described in [23]. Briefly, the method starts with defining the network's nodes as small regions of interest in the native space grey matter segmentations that correspond to 3×3×3 voxel cubes. These cubes keep the three dimensional structure of the cortex intact and so geometrical information is used in addition to the grey matter values in the voxels. The similarity between all the nodes in the network was determined with the correlation coefficient, as this metric is simple to understand and implement, while at the same time fast to compute [39]–[42]. The numerator of the correlation coefficient r_jm_ between cubes v_j_ and v_m_ calculates the sum over the product of the differences between the cubes' values at each voxel location i = 1, 2, … n for n voxels (after subtraction of the cubes' average values, respectively v__j_ and v_m__ ). The denominator of the correlation coefficient is the product of the cubes' standard deviations:



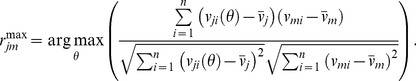
(1)


Given that the cortex is a curved object, two similar cubes could be located at an angle from each other, which could decrease their similarity value. Therefore, the maximum correlation value was computed over different rotations of the seed cube. Regions with zero variance in grey matter values were excluded (average across all subjects <0.01%), because in these cases the correlation coefficient is undefined.

Next, the similarity matrices were binarised to construct unweighted and undirected graphs after determining a threshold for each individual graph with a permutation based method to ensure a similar chance of including 5% (*SD* = 0.002) spurious correlations for all individuals (see [40]). Only positive similarity values survived this threshold. The graphs were undirected because causality cannot be inferred from correlations. Although continuous weights would contain the most information [43], [44], the present study assessed only the basic network topology and therefore the networks were binarised for simplicity.

Note that in the present study the term “connectivity” is used to indicate when two nodes show high statistical similarity, which can exist in the absence of axonal connectivity.

### Graph properties

In order to quantitatively describe the single-subject graphs and to maximise comparability of our results with those reported by previous graph studies in AD, we assessed the following basic network properties: the size of networks, the connectivity density (i.e., the proportion of existing connections to the maximum number of possible connections), the degree, the characteristic path length (i.e., the minimum number of edges between any pair of nodes), the clustering coefficient [45] and the betweenness centrality [46]. All these properties were measured at local (i.e., single nodes) and global (i.e., averaged across nodes) scales. In addition, the small world coefficient [45], [47] was computed by normalising the average clustering coefficient and characteristic path length of each graph with those averaged from 20 randomised reference graphs of identical size and degree distribution [48]. Normalised clustering coefficient was denoted as γ, and normalised characteristic path length was denoted as λ. A network has the small-world property when γ/λ>1, suggesting that its topology is different from that of a random graph [45], [47]. This connectivity architecture is thought to be efficient as clusters can be regarded as specialised units in a network that can exchange information via sparse connections between them. We found that 20 random reference graphs were sufficient to produce stable results (range *SD* values across subjects, γ: min = 0.0002, max = 0.0006; λ: min = 7.26×10^−7^, max = 4.19×10^−6^; σ: min = 0.0002, max = 0.0005).

Another feature of brain graphs is the existence of nodes with a central role in a network, i.e., “hubs”. The existence of hubs in a network decreases the characteristic path length and renders graphs more resilient towards random attack of nodes and/or edges (e.g., [49]). However, this also makes them the weakest points of networks. Hubs have been proposed to be specifically targeted in AD (e.g., [33], [35]). Here, hubs were identified as nodes with a higher than one standard deviation above the average betweenness centrality. More technical details of these measurements and their interpretation have been extensively discussed elsewhere (e.g., [15], [17], [44], [50]–[52]). Network extraction and computation of their properties were all computed with in house software that was developed in Matlab v7.12.0.635 and modified scripts from the Brain Connectivity Toolbox (www.brain-connectivity-toolbox.net, [44]).

### Statistical comparisons

Statistical comparisons were performed in R version 2.15.0 (30-03-2012). Lilliefors tests from the R package “nortest” indicated a significant departure from normality of the distributions for some of the network properties. Log-transformation proved unsuccessful in rendering the data normal, and therefore we used a rank transformation of network properties values so that we could use parametric analysis methods [53], [54]. Ties were replaced by average ranks.

It has been demonstrated that differences in graph size and connectivity density influence other network properties [55], [56]. In the present study the distributions of the graph defining properties (i.e., size, average degree and connectivity density) and also the property-interrelationships showed close correspondence between the groups, thus allowing for comparisons of other network properties (see Table S1 in File S1).

Group comparisons of the remaining graph properties were tested with ANCOVAs using grey matter volume, age and gender as covariates. The groups had similar equality of variance for all graph properties as measured with the Levene's test (all p>0.05). In order to reduce dimensionality and aid comparability among graphs but also with other studies, local graph properties were summarised by averaging these across the cubes within each of the 90 anatomical areas that were defined in individual subjects by means of the Automated Anatomical Labelling atlas (AAL, [57]) using the statistical parametric mapping toolbox of SPM8 (IBASPM). This toolbox was also used to obtain measurements of total grey matter volume and local volumes of the AAL regions. Group differences in betweenness centrality were tested for each anatomical area with ANCOVAs including regional volume, age and gender as covariates. Relationships between global and local graph properties and MMSE scores were determined with Pearson's correlations. Using forward regression and starting with a model including only global volume and hippocampal volume as predictors of MMSE, we assessed how prediction improved after adding the graph properties that showed the strongest correlation with MMSE. Finally, where appropriate we computed *p* values using the False Discovery Rate (FDR, [58], [59]).

## Results

### Subject characteristics

**Figure 1:**
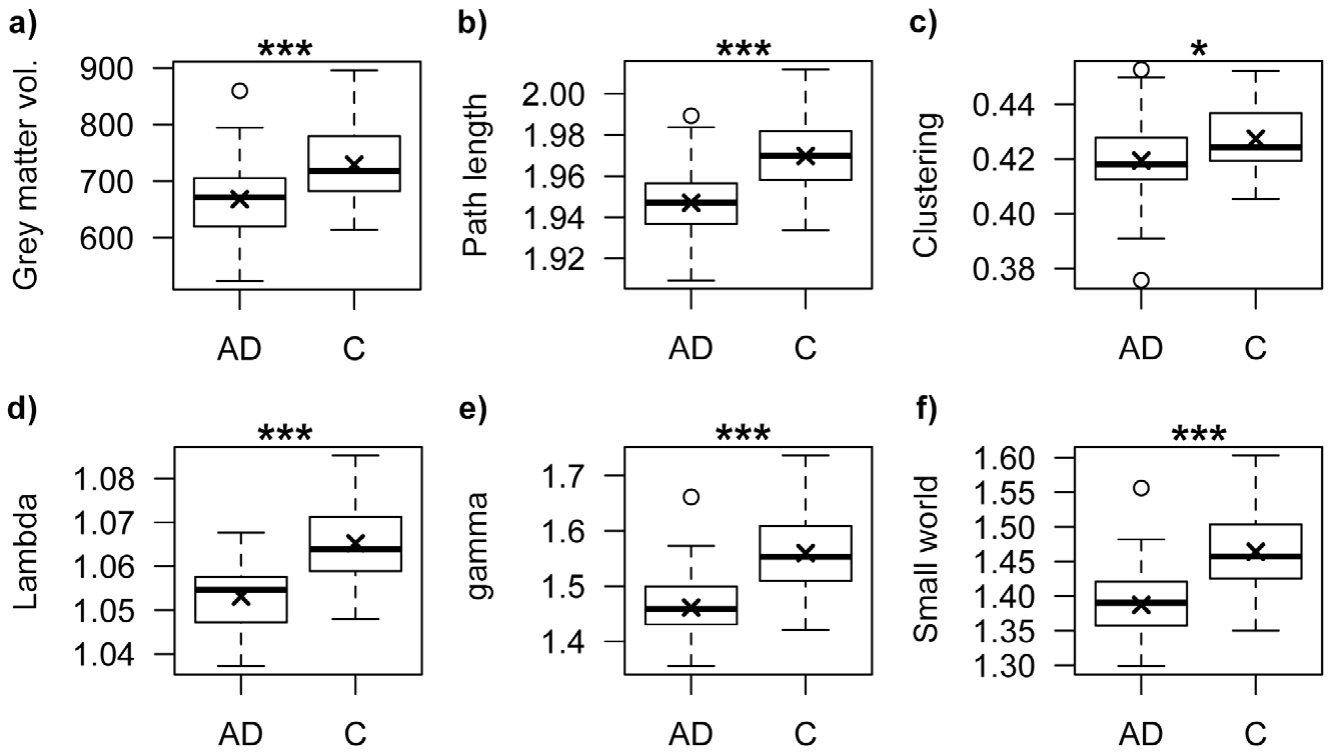
Box plots visualising differences in the distributions of global graph property values between Alzheimer's disease patients (AD) and control subjects (C). Box plots show the distributions of: a) grey matter volume: F (1, 74) = 13.78, *p* = 0.0004, b) the average path length: F (1, 71) = 19.62, *p* = 3.35×10^−5^, c) the average clustering coefficient: F (1, 71) = 4.58, *p* = 0.04, d) average λ: F(1, 71) = 33.30, *p* = 1.91×10^−7^, e) average γ: F (1, 71) = 23.45, *p* = 7.25×10^−6^ and f) the small world property F (1, 71) = 19.50, p = 3.53×10^−5^. Middle line indicates the median value, the cross indicates the mean. * = *p*<0.05; *** = *p*<0.001, significance of ANCOVAs after adjustment of total volume, age and gender.

The main subject characteristics are summarised in Table 1. The groups were successfully matched on gender (both groups contained 19 female and 19 male subjects) and did not differ in age (mean age AD: 72.06±4.32 years; mean age controls: 71.91±4.32). As expected, the AD patients scored significantly lower on the MMSE than control subjects (*p* = 7.65×10^−15^). Furthermore, global grey matter volume was significantly lower in AD (*p* = 0.0004; see Figure 1a). All single-subject grey matter graphs were fully connected through on average 8683 nodes (*SD* = 545) and an average connectivity density of 15% (*SD* = 0.74) across all subjects.

**Table 1:** Subject characteristics.

	Group		
	AD	C	p-value
Sample size	38	38	na
Age	72.06±4.32	71.91±4.32	≈1
Gender (M/F)	19/19	19/19	1
average MMSE+/−SD	19.65±5.42	27.67±2.20	7.65×10^−15^
average grey matter volume in mm^3^	668.23±74.62	729.33±65.04	0.0004

*AD* is Alzheimer's disease patients, *C* is control group, *M* is male, *F* is female, *MMSE* is Mini-mental state examination, *SD* is standard deviation, *mm^3^* is cubic millimetre, *na* is not applicable.

### Alterations of global network properties in Alzheimer's disease

All single-subject grey matter graphs in both AD and control groups had higher average clustering than random reference networks (i.e., γ>1. Range γ across all individuals: min = 1.35, max = 1.73) and similar average path length (i.e., λ≈1. Range λ across all individuals: min = 1.04, max = 1.08), indicating that all networks had a small world topology (i.e., γ/λ>1. Range across all individuals: min = 1.30, max = 1.60). However, this topology was altered in AD. All full ANCOVA's models were significant after correction for multiple hypotheses testing with FDR. The graphs from AD patients were characterised by a decreased characteristic path length (*F* (1, 71) = 19.62, *p* = 3.35×10^−5^; Figure 1b) and decreased clustering coefficient (*F* (1, 71) = 4.58, *p* = 0.04; Figure 1c) in comparison to graphs from control subjects. Furthermore, Figures 1e to f show that the small world properties were significantly lower in AD (λ: *F* (1, 71) = 33.30, *p* = 1.91×10^−7^; γ: *F* (1, 71) = 23.45, *p* = 7.25×10^−6^; the small world property: *F* (1, 71) = 19.50, *p* = 3.53×10^−5^). The betweenness centrality did not differ between the groups (*F* (1, 71) = 0.06, *p* = 0.81). Taken together, these results suggest that AD networks move towards a more random topology. Because these graph properties were corrected for global grey matter volume, it is unlikely these results simply reflect cortical atrophy.

### Local graph disturbances in Alzheimer's disease

**Figure 2:**
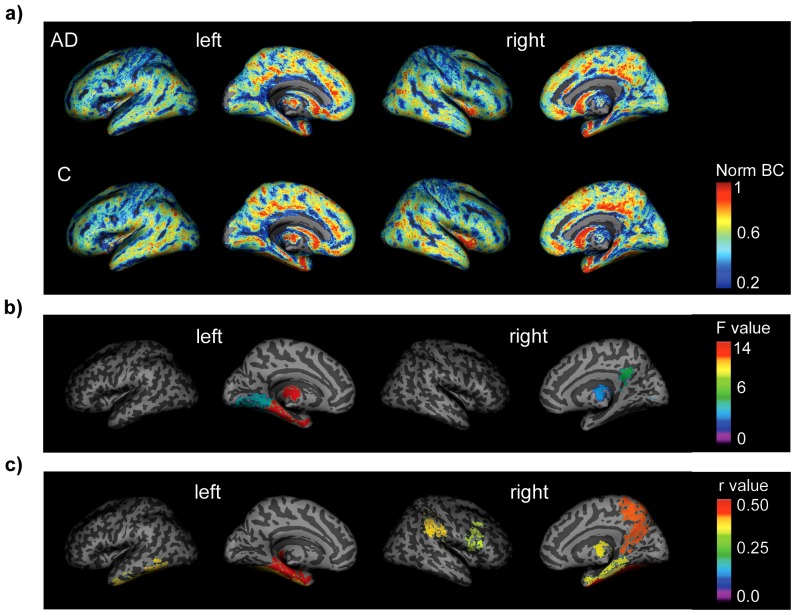
The spatial distribution of betweenness centrality (BC) values across the cortex, group differences in local BC and local path length. a) BC values were averaged over subjects in the Alzheimer's disease (AD, upper panel) and control (C, lower panel) groups. After individual maps were normalised for graph size they were warped into MNI space where they were averaged for each group. Average maps were normalised again to values between 0–1. The spatial distribution of the unnormalised BC values showed a strong correlation between the groups (ρ = 0.74, p<2.2×10^−16^). b) Surface plots of the F values of the AAL regions that showed a significantly decreased average BC value after correction for local grey matter volume, gender and age. c) Surface plots of the 20 AAL regions that showed a significant (p<0.05) correlation between local path length (L) and mini-mental state examination (MMSE) scores (See Figure S1 in File S1 for the scatter plots of all significant correlations).

The betweenness centrality is commonly used to assess which nodes are “hubs”, indicating that they are more centrally connected relative to the other nodes in a network. The groups showed a large difference in uncorrected BC (*F* (1, 74) = 8.18, *p* = 0.006), complicating the interpretation of the comparison of a relative hub definition. As an alternative, we compared regional BC between the groups. Figure 2a shows the spatial distribution of local BC values, after normalization for graph size for each group. We further investigated whether groups differed at a regional level in BC. Twenty-nine of the 90 ANCOVAs were significant after correction for multiple hypotheses testing with FDR. Figure 2b shows specific anatomical areas where the betweenness centrality was significantly reduced in the AD group after correcting for local grey matter: the right posterior cingulate gyrus (*F* (1, 71) = 6.25, *p* = 0.01), left parahippocampal gyrus (*F* (1, 71) = 13.83, *p* = 0.0004), left lingual gyrus (*F* (1, 71) = 5.13, *p* = 0.03) and bilateral thalami (left *F*(1, 71) = 13.12, *p* = 0.0005; right *F* (1, 71) = 4.07, *p* = 0.05). The left parahippocampal gyrus, left lingual gyrus and bilateral thalami of the AD group also showed decreases in clustering coefficient and path length (See Table S6 in File S1. See Figure S2 in File S1 for an overview of local graph properties distributions for each group).

### Associating graph alterations with symptom severity

**Figure 3:**
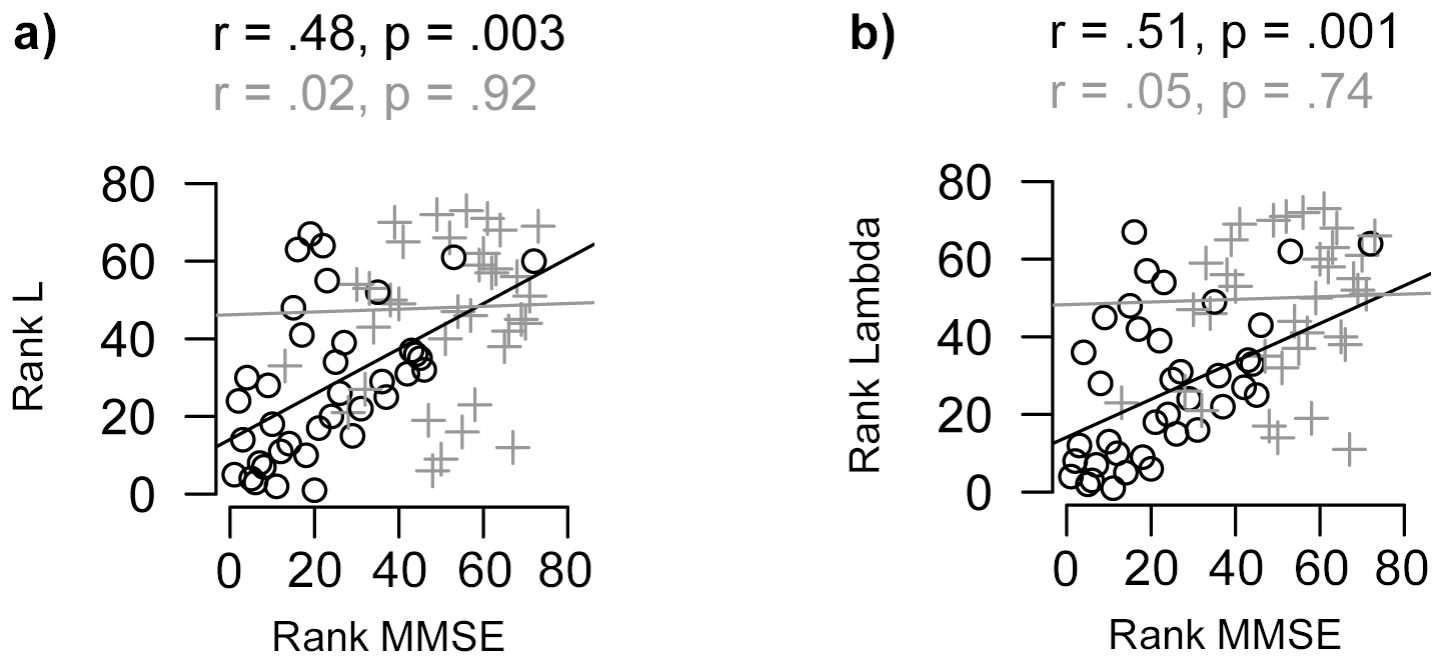
Scatter plots of rank-transformed scores on the mini-mental state examination (MMSE) with global path length L and λ. Correlations between MMSE and L (a) and λ (norm. L.; b) for the AD (black circles) and control group (dark grey plus signs). Note that global grey matter volume, graph size and connectivity density were unrelated to MMSE scores (resp. r = −0.18, *p* = 0.28; r = −0.18, *p* = 0.29; r = −0.06, *p* = 0.73), nor were such relationships found with local grey matter in any of the AAL regions.

We further scrutinised the relationships between graph alterations and general cognitive decline in AD as measured with the MMSE (available for 73 of the 76 subjects). The average characteristic path length was strongly related to MMSE scores (*r* = 0.48, *p* = 0.003, *p_FDR_* = 0.01, 95% confidence interval = [0.18–0.70]; Figure 3a left), as was λ (*r* = 0.51 *p* = 0.001, *p_FDR_* = 0.01, 95% confidence interval = [0.23–0.72]; Figure 3a right). In some AAL regions, similar associations between local path length and MMSE scores were found (see Table 2 and Figure S1 in File S1): The characteristic path length of the left parahippocampal gyrus showed the strongest relationship with MMSE scores, followed by the left hippocampus, right fusiform gyrus and right precuneus. However none of these survived correction for multiple comparisons with FDR.

**Table 2:** Spearman's rank correlations (ρ) between MMSE scores and local path length after adjustment for local grey matter volume, gender and age.

				95% CI	
Cortical Region	ρ	p	p_FDR_	min	max
L parahippocampal gyrus	0.50	0.0016	0.15	0.21	0.71
R fusiform gyrus	0.45	0.0055	0.19	0.14	0.67
L hippocampus	0.44	0.0063	0.19	0.14	0.67
R precuneus	0.40	0.0139	0.31	0.09	0.64
R supramarginal gyrus	0.37	0.0233	0.31	0.05	0.62
R Heschl's gyrus	0.37	0.0253	0.31	0.05	0.62
L fusiform gyrus	0.36	0.0282	0.31	0.04	0.61
R parahippocampal gyrus	0.36	0.0297	0.31	0.04	0.61
L inferior temporal gyrus	0.35	0.0335	0.31	0.03	0.61
R thalamus	0.35	0.0348	0.31	0.03	0.60
R inferior occipital gyrus	0.33	0.0460	0.32	0.01	0.59
L middle orbitofrontal gyrus	0.33	0.0468	0.32	0.01	0.59
*CI* is confidence interval					

Ties were averaged.

Next, we examined whether these graph properties could explain more variance in MMSE scores than global and hippocampal grey matter volume. Table 3 shows that a model including λ and path length in the left parahippocampal gyrus explained 32% of the variance in MMSE scores (*adjusted R^2^* = 0.32), which was a significant improvement (*p* = 0.004) when compared to the model including only global grey matter and hippocampal volume (*adjusted R^2^* = 0.09). Adding the hippocampal path length to the model did not lead to further improvements (*p* = 0.79). To conclude, within the AD group more random graphs were associated with worse cognitive dysfunction, supporting the clinical relevance of single-subject grey matter graphs.

**Table 3:** Comparing regression models to explain variance in Mini-Mental State Examination scores within the Alzheimer's disease group.

Model description		Adj. R^2^	F	p
Model 1	Global GM volume+GM hippocampus	0.09		
Model 2	Model 1+λ	0.20	5.87	0.02
Model 3	Model 2+path length of left parahippocampal gyrus	0.32	6.59	0.02*
			6.72	0.004**

*GM* is grey matter volume, * is comparison between Model 2 and Model 3, ** is comparison between Model 1 and Model 3.

Ties in rank scores where averaged.

## Discussion

The present study demonstrated AD-related alterations in *single-subject* grey matter graphs, using a recently developed method to describe patterns of intracortical similarities from MRI data. One of the main findings was that the topology of AD graphs was more random in comparison to control subjects. In addition, decreases in nodal centrality were found in specific anatomical areas known to be involved in AD, suggesting that the contributions of cortical areas to global disruptions are heterogeneously distributed across anatomical areas. Importantly, by using single-subject grey matter graph measurements we were able to demonstrate a relationship between graph alterations and general cognitive decline at both whole brain level and in specific cortical areas. These findings could not be explained by volumetric differences. The present results support the clinical relevance of single-subject structural brain graphs.

### Interpretation of intracortical similarities

At this point the biological mechanisms underlying intracortical similarities are still unknown and we can only speculate about the causes of AD related alterations in these patterns. Intracortical similarities might be induced by functional coherence of cortical areas that has been demonstrated to cause coordinated changes of cortical structures within individuals [60] and across individuals in groups [24], [61]. This hypothesis may explain why areas with many connections were distributed across areas known to be involved in the “default mode network”: regions that show more functional synchronisation during rest than during tasks [62] (see File S1). Notably, evidence exists that the default mode network is altered in AD (e.g., [63], [64]), and even shows alterations in young individuals who are at an increased genetic risk for AD [65]–[67]. The present results support this hypothesis, as local graph disruptions show overlap with this network.

In addition to functional coactivation, intracortical similarities might arise as a result of axonal connectivity that can influence morphological measurements of the cortex [68], [69], [70]. A recent study has reported that about 40% of cortical thickness correlations assessed at a group-level converged with group-averaged DTI traced tracts [71]. Furthermore, cortical morphology might also arise as a result of mutually trophic influences [72], [73].

Disruptions in any of these processes are likely to contribute to changes in patterns of intracortical similarity that are related to AD.

### Grey matter network alterations in Alzheimer's disease

The presently investigated single-subject graphs were characterised by a more random topology in AD and this was indicated by a decreased path length, clustering coefficient and small world coefficient (including both γ and λ). Most of these results are in line with previous functional studies that also have demonstrated more random topologies in AD [29]–[31], which is suggestive of an association between functional and morphological disruptions.

Furthermore, local measurements were heterogeneously affected, indicating that specific areas are targeted in the disease. The areas that showed decreased betweenness centrality in AD included the posterior cingulate, the parahippocampal and lingual gyri. These structures have previously been reported to be altered in group-based structural graphs [26], [28] as well as in functional graphs [30], [31]. Moreover, the parahippocampal gyrus and posterior cingulate areas are part of the Papez circuit that is involved in memory [74]. The posterior cingulate fasciculus that connects these areas has been reported to be affected in AD [75].

Another important finding of the present study was that decreased characteristic path length in medial temporal areas, including the hippocampus, but also in other areas such as the right precuneus was related to worse cognitive performance and that these relationships could not be explained by differences in local volumetric measurements. These results imply that graph measurements have the potential to monitor disease progression, which is important for the development of new treatments.

Interestingly, some of the areas where we found graph disruptions, such as the posterior cingulate cortex and precuneus, are known to be highly interconnected in both functional and anatomical graphs [76], [77] and are also part of the default mode network [78], [79]. Previous studies have reported functional disruptions in these areas and metabolic disruptions in AD [67]. Evidence has also been reported of a positive association between the amount of amyloid plaques and increased functional connectivity [33], suggesting that functional hubs are more vulnerable to AD pathology. A recent simulation study demonstrated that these areas also show more resting-activity and are therefore specifically at risk for activity driven degeneration and that such degeneration rendered topologies more random [80]. The finding that grey matter graphs also become more random might reflect functional dysconnectivity in AD. Future research is needed to further investigate this relationship.

### Remaining issues and conclusion

A priori differences in network size and/or connectivity density complicate subsequent graph comparisons, because these properties influence other network property values [55], [56], [81]–[83]. Therefore most studies use anatomical templates to ensure the same number of nodes and enforce identical connectivity density to facilitate comparisons. However, cortical structure is highly variable across individuals and therefore the same region defined in different people with an anatomical template could consist of different gyri and sulci patterns [84], or might not even exist due to atrophy. In addition, results from recent studies suggest that AD is characterised by a loss of connectivity and is therefore a dysconnectivity disease [85]. Enforcing a similar connectivity density between groups will therefore result in the inclusion of more spurious connections in AD graphs, rendering their topology more random. For these reasons the graphs in the current study were analysed in their native space and binarised such as to ensure a similar chance of including spurious edges. The groups showed similar distributions of size and connectivity density and similar interrelationships between the graph properties, supporting that the present results reflect structural graph disruptions due to Alzheimer's disease. In addition, global volume, graph size and connectivity density were unrelated to disease severity, while altered graph properties showed strong relationships further underlining the clinical importance of single-subject graphs.

The single-subject graphs of our previous study [23] had a higher connectivity density than those presently investigated, using the same procedure to threshold the graphs. This may have been caused by differences in scanner strength (1.5T vs 3T) and/or sample composition (34.80+/−8.23 years vs 71.91+/−4.32 years). The comparison of scans acquired at different strengths and/or scanners is a general difficult issue in neuroimaging research and a detailed investigation of this issue is outside the scope of the present study.

We found strong relationships between global cognitive functioning as measured by the MMSE and altered single-subject graph properties, that were absent with volumetric measurements. However, a limitation of the MMSE is that it is mostly a clinical screening tool and should not be regarded as an elaborate neuropsychological assessment. Future research is needed to examine whether altered graph properties are related to dysfunction in specific cognitive domains. In addition, the diagnostic potential of these graphs should be further investigated using sophisticated classification algorithms such as support vector machines (e.g., [27], [86], [87]).

The present finding of a decreased average characteristic path length in AD was unexpected because other structural studies have associated AD with increased characteristic path lengths [26], [28]. To better understand these seemingly discrepant results with previous group-based studies we constructed group-based graphs for both AD and control groups as previously described in [28] and explored how the use of different threshold settings (i.e., enforce equal connectivity density or equal probability of spurious connections) influence group differences (see File S1 and Tables S2, S3, S4 and S5 therein for a full description of these exploratory analysis). Our main finding was that path length can be increased as a consequence of disconnected nodes, while at the same time λ is decreased in AD suggesting a move towards more random graphs. In addition, we found for all thresholding procedures an increased clustering coefficient in AD, which was associated with a decreased γ, also indicative of a more random graph topology. Most importantly, these exploratory results demonstrate that the traditionally used thresholding-method to enforce equal connectivity density on graphs can introduce differences between the groups in the number of connected areas, and also in the average weight of the edges (which indicates that groups differed in the amount of included spurious connections). Additionally, the weights in the group-based AD connectivity matrix were on average higher than those of controls (which is likely to be related with the higher clustering coefficient value). All single-subject graphs in the current study were fully connected and did not differ in the average weight of included edges, suggesting that these factors cannot explain group differences in other graph properties.

Our results show closer resemblance to those reported by functional graph studies [29]–[32]. A recent study that investigated EEG graph topology during healthy ageing implied a developmental curve that follows an inverted U-shaped trajectory [88] where graph topologies in young children (<20 years) and elderly people (>55 years) were more random than those in adulthood. In addition, more random topologies in elderly subjects were associated with reductions of grey matter volume. Therefore, variability in group differences reported across studies might reflect that graphs were investigated at different points on this developmental curve. The present results support this hypothesis, since in AD shorter normalised path lengths were associated with more cognitive impairment (also found by [32]; although also see: [89], [90]). Furthermore, we found that a shorter characteristic path length in the precuneus of control subjects was associated with better cognitive functioning (see File S1), which is in line with the general assumption that shorter path lengths are associated with more efficient networks [91]–[93]. Future research is needed to further investigate how graph alterations are related to changes of cognitive functioning during the development of AD.

The current approach could be used to further investigate why the spatial pattern of plaques in AD does not show a clear correspondence with tangles and atrophy patterns: Possibly, coordinated changes in cortical structure in AD are caused by disrupted functional dynamics due to plaques and tangles. Future research will further investigate this question by combining functional and structural graphs within subjects.

Finally, the control group in the present study comprised people who entered the clinic with memory complaints, but were found to have no objective cognitive disorder after testing. It cannot be ruled out that these people might experience a very early stage of the disease, as they have a higher chance to develop AD [94]. If so, the present results might be underestimated, but their clinical relevance is increased, as this group is more representative of people who visit memory clinics.

In conclusion, this study demonstrated evidence of the clinical value of single-subject grey matter graphs, which provide a concise quantitative description of disruptions in coordinated morphological patterns of entire brains. The present results contribute to a better understanding of the relationship between structural changes and cognitive dysfunction in Alzheimer's disease.

## Supporting Information

File S1 **Supporting information.**
(DOC)
