# Correction: Single-Subject Grey Matter Graphs in Alzheimer's Disease

**DOI:** 10.1371/annotation/6a2e6405-ce1d-49e0-a88c-0017c680d597

**Published:** 2013-09-09

**Authors:** Betty M. Tijms, Christiane Möller, Hugo Vrenken, Alle Meije Wink, Willem de Haan, Wiesje M. van der Flier, Cornelis J. Stam, Philip Scheltens, Frederik Barkhof

In the Author Contributions statement, the first author (BMT) should be listed as one of the authors who interpreted the data.

