# Supplementary material for: Single-Subject Grey Matter Graphs in Alzheimer's Disease
Source: PLoS One. 2013 Mar 11;8(3):e58921. doi: 10.1371/journal.pone.0058921 (PMC3594199; doi:10.1371/journal.pone.0058921)
Supplement: File S1 — Supporting information. (DOC) [file pone.0058921.s001.doc]

**Supporting Information S1**

**Graph comparability**

We tested whether groups showed a priori differences in graph size and connectivity density, since these influence other network measurements (e.g., [1, 2]). The AD and control groups had graphs of similar size (F (1, 74) = 0.62, *p* = 0.43), average degree (F (1, 74) = 0.45, *p* = 0.50), connectivity density (F (1, 74) = 0.15, *p* = 0.70) and a similar chance of spurious connections in their networks (F (1, 74) = 0.06, *p* = 0.81). Nodes with a degree higher than one standard deviation above the mean (i.e., ‘degree hubs’) were distributed along the medial surface of the cortex, in line with our previous findings, and this distribution was similar for the two groups (H = 0.006, *p* = 0.94). Furthermore, the groups showed similar interrelationships between the graph properties as measured with Pearson’s correlations (see Table S1). Since the distributions of the graph defining properties and also the property-interrelationships were similar for both groups, we could proceed with group comparisons of all other graph properties.

| **Table S1**  Pairwise correlations between graph properties across people, for the control (C) and Alzheimer’s disease (AD) groups. | | | | | | | | | | | | | | |
| --- | --- | --- | --- | --- | --- | --- | --- | --- | --- | --- | --- | --- | --- | --- |
|  | k |  | V |  | L |  | CC |  | SW |  | CD |  | BC |  |
|  | C | AD | C | AD | C | AD | C | AD | C | AD | C | AD | C | AD |
| k | 1 | 1 | 0.71*** | 0.83*** | -0.37* | -0.56*** | 0.49*** | 0.56*** | -0.02 | 0.19 | 0.55** | 0.69*** | 0.47** | 0.72*** |
| V |  |  | 1 | 1 | -0.08 | -0.32 | -0.09 | 0.17 | 0.19 | 0.28 | -0.11 | 0.25 | 0.87*** | 0.96*** |
| L |  |  |  |  | 1 | 1 | -0.01 | -0.29 | **0.84***** | **0.36** | -0.48** | -0.60*** | 0.35 | -0.08 |
| CC |  |  |  |  |  |  | 1 | 1 | 0.08 | 0.41* | 0.88*** | 0.88*** | -0.16 | 0.10 |
| SW |  |  |  |  | Z = 3.50* |  |  | 1 | 1 | -0.29 | 0.16 | 0.52** | 0.39 |  |
| CD |  |  |  |  |  |  |  |  |  |  | 1 | 1 | -0.33 | 0.11 |
| BC |  |  |  |  |  |  |  |  |  |  |  |  | 1 | 1 |
| *** p_FDR_ < 0.001; ** p_FDR_ < 0.01; * p_FDR_ < 0.05  *k* is mean degree, *V* is mean number of nodes, *L* is mean characteristic path length, *CC* clustering coefficient, *SW* is small world, *CD* is connectivity density, *BC* is betweenness centrality. | | | | | | | | | | | | | | |

**Group-wise volumetric graph explorations with current sample.**

***Introduction***

The single-subject grey matter graphs investigated in the current study showed a decreased average path length in AD, while other studies (across different imaging modalities) have reported increased path lengths in AD. However, raw path length is dependent on the connectivity density of graphs. Since previous studies have compared path length between AD and control groups after enforcing a similar connectivity density, the increase in path length might also reflect differences in the number of spurious and/or absent connections between groups. Therefore comparing λ (i.e., normalised path lengths) might be more informative of ‘true’ graph differences between groups.

Different studies across imaging modalities have reported both increased and decreased λ in AD. In addition, it remains unknown how groups differ in λ and γ in group based grey matter graph studies.

In order to gain more insight into these apparently discrepant results of the current single-subject approach with those from group-based studies, we explored in this section whether differences in group-based graph properties change under different thresholding conditions.

***Methods***

Group-based grey matter graphs were constructed by assessing the correlations of grey matter volume between 90 AAL regions across individuals (using a procedure as explained in [3]). We first performed a regression of the volumetric measurements against global grey matter volume, age and sex in order to account for differences between subjects in these covariates. The resulting residuals were then used to assess the interregional correlations for each group.

| **Table S2** Different thresholding conditions for group-based grey matter volume graphs | |
| --- | --- |
| ID | Description of thresholding procedure |
| 1 | Binary , 15% conn dens absolute values |
| 2 | Binary , 15% conn dens positive values |
| 3 | Fully weighted, absolute values |
| 4 | Fully weighted, positive values |
| 5 | Binary, same p_FDR = 0.05, absolute |
| 6 | Binary, same p_FDR = 0.05, positive |

In order to investigate how thresholding affects graph properties we computed graph properties for three different types of graphs: 1. Thresholded such that both graphs had a connectivity density of 15% (corresponding to the average connectivity density of the single-subject graphs). This means that both groups can have different thresholds. 2. A fully weighted graph, 3. Thresholded such that graphs only contain connections that survive correction for multiple comparisons with FDR. This means that graphs from both groups can differ in their connectivity density. To further disentangle the contribution of negative weights, we performed all analysis over both the absolute connectivity matrix and a connectivity matrix including only positive correlations. All thresholding conditions explored here are listed in Table S2.

For each graph we determined the average absolute weights, average of weights > threshold, the connectivity density, and the number of disconnected nodes. In addition we computed the following graph properties: the average characteristic path length, average clustering coefficient. λ, γ and the small world coefficient (γ / λ).

The average path length and clustering coefficient were normalised by the path length and clustering coefficient that was averaged across 100 random reference graphs that kept intact the degree/strength distribution in order to compute λ and γ.

***Results***

*A priori differences in correlation matrices*

Table S3 summarises the a priori existing differences in correlation values found. Average raw, absolute and positive correlations were increased in both single-subject and group-based graphs in AD when compared to controls. In addition both single-subject and group-based graphs in AD contained a higher percentage of positive correlations than control graphs. Both single-subject and group-based graphs in AD showed a lower percentage of negative correlations than controls. Finally, single-subject grey matter graphs in AD showed on average weaker negative correlations than controls, while group-based graphs in AD contained on average stronger negative correlations.

Note that these differences were qualitatively larger between AD and controls for group-based graphs than for single subject-graphs.

| **Table S3** General weight information | | |  |  |
| --- | --- | --- | --- | --- |
|  | Group based graph  size = 90 | | Single subject graphs  average size = 8683 | |
|  | Group | | Group | |
| Type of average | AD | SMC | AD | SMC |
| Average over all raw connections | **0.2706** | 0.1587 | **0.3975** | 0.3924 |
| Average over all absolute connections | **0.3040** | 0.2110 | **0.3976** | 0.3926 |
| Average over all positive connections | **0.3358** | 0.2474 | **0.3982** | 0.3933 |
| % Positive connections | **86.52** | 75.56 | **99.83** | 99.81 |
| Average over all negative connections | **-0.1253** | -0.1081 | -0.0515 | **-0.0542** |
| % Negative connections | 13.48 | **24.44** | 0.1709 | **0.1903** |
| The largest value is indicated with **bold** typeface. | | | | |

| **Table S4** Differences between Alzheimer’s patients and controls in *unnormalised* graph properties of group-derived grey matter volume graphs under different thresholding conditions. | | | | | | | | | | | | |
| --- | --- | --- | --- | --- | --- | --- | --- | --- | --- | --- | --- | --- |
|  | Graph property | | | | | | | | | | | |
|  | L | | CC | | 1/E | | % existing connections | | Weight > t | | # disconnected nodes | |
| Thresholding condition | AD | C | AD | C | AD | C | AD | C | AD | C | AD | C |
| *Present study* | *1.95* | ***1.97*** | *0.42* | ***0.43*** | na | na | *15* | *15* | ***0.67*** | *0.66* | *0* | *0* |
| 1 | **10.61** | 2.30 | **0.53** | 0.50 | 6.60 | **6.62** | 15 | 15 | **0.63** | 0.51 | 3 | 0 |
| 2 | **13.81** | 2.33 | **0.53** | 0.51 | 6.60 | **6.62** | 15 | 15 | **0.63** | 0.52 | 3 | 0 |
| 3 | 3.14 | **3.94** | **0.27** | 0.17 | 2.78 | **3.54** | 100 | 100 | **0.27** | 0.16 | 0 | 0 |
| 4 | 13.28 | **76.24** | **0.28** | 0.17 | 3.44 | **5.35** | **87** | 76 | **0.34** | 0.25 | 0 | 0 |
| 5 | 1.82 | **6.19** | **0.60** | 0.51 | 3.15 | **9.54** | **32** | 10 | 0.53 | **0.56** | 0 | 0 |
| 6 | 1.90 | **6.21** | **0.60** | 0.52 | 3.18 | **9.56** | **31** | 10 | 0.54 | **0.56** | 0 | 0 |
| See Table S2 for description of Thresholding conditions. *L* is path length, *CC* is clustering coefficient, *1/E* is inversed global efficiency, *t* = threshold, *#* is number of. | | | | | | | | | | | | |

| **Table S5** Differences between Alzheimer’s patients and controls in *normalised* graph properties of group-derived grey matter volume graphs under different thresholding conditions. | | | | | | | | | | | | |
| --- | --- | --- | --- | --- | --- | --- | --- | --- | --- | --- | --- | --- |
|  | Graph property | | | | | | | | | | | |
|  | λ | | γ | | σ | | % existing connections | | Weight > t | | # disconnected nodes | |
| Network type | AD | C | AD | C | AD | C | AD | C | AD | C | AD | C |
| *Present study* | 1.05 | 1.06 | 1.46 | **1.56** | *1.39* | *1.46* | *15* | *15* | ***0.67*** | *0.66* | *0* | *0* |
| 1 | 1.04 | **1.14** | 2.00 | **2.51** | 1.93 | **2.21** | 15 | 15 | **0.63** | 0.51 | 3 | 0 |
| 2 | **1.35** | 1.15 | 2.02 | **2.57** | 1.49 | **2.23** | 15 | 15 | **0.63** | 0.52 | 3 | 0 |
| 3 | 1.13 | **1.16** | 1.0003 | **1.001** | **0.89** | 0.87 | 100 | 100 | **0.27** | 0.16 | 0 | 0 |
| 4 | 4.58 | **22.09** | 1.01 | **1.07** | **0.22** | 0.05 | **87** | 76 | **0.34** | 0.25 | 0 | 0 |
| 5 | 1.06 | **2.74** | 1.28 | **3.83** | 1.21 | **1.40** | **32** | 10 | 0.53 | **0.56** | 0 | 0 |
| 6 | 1.10 | **2.74** | 1.28 | **3.79** | 1.17 | **1.38** | **31** | 10 | 0.54 | **0.56** | 0 | 0 |
| See Table S2 for description of Thresholding conditions. *λ* is normalized path length, *γ* is normalized clustering coefficient, σ is small world coefficient, *t* = threshold, *#* is number of. | | | | | | | | | | | | |

*Characterising weights and connectivity density in thresholded correlation matrices*

The fourth, fifth and sixth columns in Supplementary Tables 4 and 5 respectively show the average weight of the edges included, the connectivity density and the number of disconnected nodes in the connectivity matrix for AD and control graphs.

Note that our approach to threshold single-subject graphs to ensure that only connections with a correlation that reaches significance at a level of p_FDR_ < 0.05 are included, resulted in connectivity matrices with a similar connectivity density of ~ 15%, and similar average edge weight (t (1, 74) = 1.67, *p* = 0.10) for both groups.

*Path length and λ*

Raw values of path length were increased in AD when group-based graphs were forced to have the same connectivity density (Supplementary Table 4). Importantly, three nodes were disconnected from the main graph in AD when only 15% of all possible connections were included (similar to the average connectivity density of the present single subject study), while the control graph remained fully connected. Apart from one condition, λ was decreased in AD (Supplementary Table 5). Global efficiency seemed less sensitive to disconnected nodes in a network: this measure was consistently decreased in AD for all types of graphs (Supplementary Table 4), which is in line with the results of the present study (note that we inversed global efficiency to interpret this as path length). We conclude that global efficiency is less sensitive to differences in: 1. disconnected nodes, 2. connectivity density and 3. weights of included edges.

*Clustering coefficient and γ*

Raw values of the clustering coefficient were consistently larger in AD than in controls under all thresholding conditions (Supplementary Table 4), which is in contrast with our current finding of a decreased clustering coefficient in single-subject AD graphs.

Furthermore, this clustering coefficient was more like their random reference graphs in AD as indicated by a *decreased* γ for all both one thresholding condition (i.e., fully weighted, absolute values; Supplementary Table 5). This was in correspondence with the present single-subject study.

*Small world coefficient*

Across all binary matrices, the small world coefficient was decreased in AD when compared to controls (Supplementary Table 5), in accordance to the present finding. However, the small world coefficient of the weighted graphs was larger in AD. This might be explained by differences between the groups in how the random reference model is determined. Note that under these conditions the small world value of the graphs from both groups was < 1 and therefore these graphs are not small world: this can occur for increasing levels of randomness [4].

***Conclusion***

In this section we reproduced the finding of an increased path length in AD of group derived grey matter graphs as was previously reported by [5, 3]. However, increased path length in group-based graphs is likely to reflect differences in: 1. the number of connected nodes and 2. differences in the distribution of correlation values in the connectivity matrix.

When graphs were binarised by only including edges that survived correction for multiple hypothesis testing, similar to the binarisation procedure of the single-subject grey matter graphs, the group differences were in the same direction as we report in our current study.

**Exploration of all local graph properties for the AAL regions where BC was decreased.**

In this section we further explored how graph properties were affected for the AAL regions that showed a decreased BC in AD, and these results are summarised in Supplementary Table 6.

| **Table S6** Mean values ± standard deviation of the degree (d), path length (Lp) and clustering coefficient (CC) in regions that were found to have a decreased BC for Alzheimer’s patients (AD) and controls (C). | | | | |
| --- | --- | --- | --- | --- |
|  | Lp |  | CC |  |
| Region | AD | C | AD | C |
| R posterior cingulate | 1.93 ± 0.05 | 1.93 ± 0.04 | 0.43 ± 0.04 | 0.44 ± 0.04 |
| L parahippocampal | 1.98 ± 0.03 | 1.98 ± 0.03 | **0.38** ± **0.02 *** | **0.39** ± **0.02 *** |
| L lingual | **1.98** ± **0.03 *** | **1.99** ± **0.02 *** | 0.35 ± 0.03 | 0.36 ± 0.02 |
| L thalamus | **1.92** ± **0.03 *** | **1.93** ± **0.02 *** | 0.43 ± 0.02 | 0.43 ± 0.02 |
| R thalamus | **1.91** ± **0.02 *** | **1.93** ± **0.03 *** | 0.43 ± 0.02 | 0.42 ± 0.02 |
| *L* is left, *R* is right, * indicates p_model_ p_effect_ and < 0.05 uncorrected. None of the models survived for multiple comparisons with the false discovery rate procedure. | | | | |

None of the regions differed in the average degree (all p > 0.05). We further found that path length was decreased in the left lingual gyrus (F = 10.50, *p* = 0.002) and bilateral thalami (left: F = 6.64, p = 0.01; right: F = 7.09, p = 0.01). In addition we found that the clustering coefficient was decreased in the left parahippocampal gyrus (F = 5.22, p = 0.03). However, none of these ANCOVA models survived correction for multiple comparisons with the false discovery rate procedure.

The group difference of decreased path length and clustering coefficient in areas with decreased BC in AD further underline that single-subject graphs become more randomly organised in AD and that this process is specific for certain areas. We further point out that the small world coefficient and global BC were positively related across individuals, further supporting that more random graphs are associated with lower global BC (see Supplementary Table 1). In addition we found *within* single-subject graphs that L was negatively related to BC.

To summarise, we interpret our group differences as suggestive of a move towards more random networks in AD where path length is reduced due to increase of randomly placed ‘long-range’ connections, and that this subsequently leads to a decreased BC.

**Distributions of local graph properties for the 90 AAL regions.**

Figure S2 shows for each of the 90 AAL regions the distribution of local graph property values for the AD (white) and control (grey) groups.

**References**

1. van Wijk BCM, Stam CJ, Daffertshofer A (2010) Comparing brain networks of different size and connectivity density using graph theory. PLoS ONE 5: e13701. doi:10.1371/journal.pone.0013701.

2. Zalesky A, Fornito A, Harding IH, Cocchi L, Yücel M, et al. (2010) Whole-brain anatomical networks: does the choice of nodes matter? Neuroimage 50: 970–983.

3.. Yao Z, Zhang Y, Lin L, Zhou Y, Xu C, et al. (2010) Abnormal cortical networks in mild cognitive impairment and Alzheimer's disease. PLoS Comp Biol 6: e1001006. doi:10.1371/journal.pcbi.1001006.t002.

4. Latora V, Marchiori M (2001) Efficient behavior of small-world networks. Phys Rev Lett. doi:10.1103/PhysRevLett.87.198701.

5. He Y, Zhang Chen, Evans A (2008) Structural insights into aberrant topological patterns of large-scale cortical networks in Alzheimer's disease. J Neurosci 28: 4756–4766.

**Supporting Figures**

**
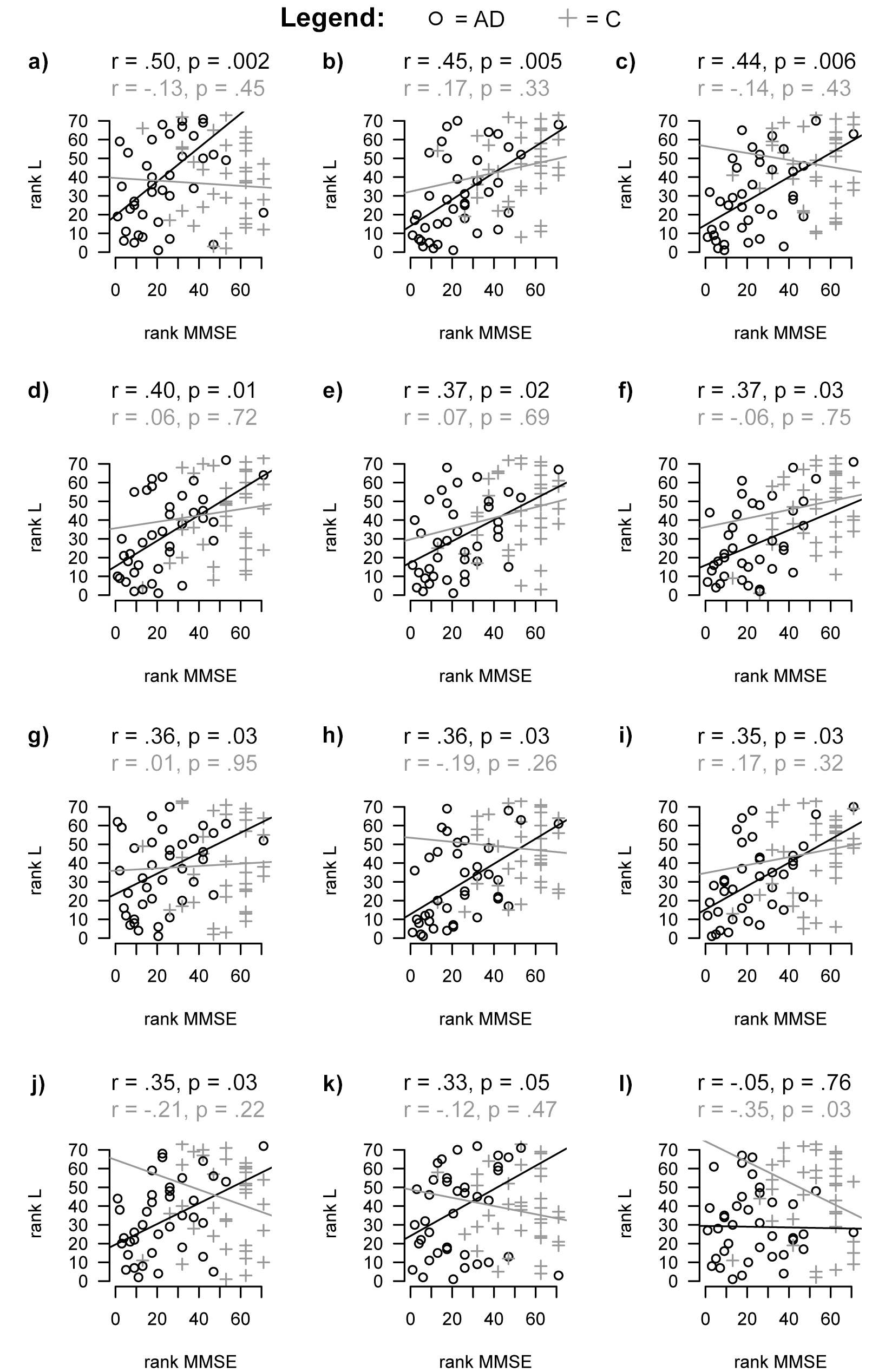
**

**Figure S1**. **Correlations between the rank-transformed scores on the mini-mental state examination (MMSE) and local path length L for the Alzheimer’s disease and control subjects.** Alzheimer’s disease patients (AD) are represented by black circles and control subjects (C) by grey plus signs. Significant correlations were found in: a) the left parahippocampal gyrus, b) right fusiform gyrus, c) left hippocampus, d) right precuneus, e) right supramarginal gyrus, f) right Heschl’s gyrus, g) right parahippocampal gyrus, h) left fusiform gyrus, i) left inferior temporal gyrus, j) right thalamus, k) left middle orbitofrontal gyrus and l) left precuneus. Note that global grey matter volume, graph size and connectivity density were unrelated to MMSE scores (resp. r = -.21, *p* = .22; r = -.26, *p* = .12; r = -.12, *p* = .50), nor were such relationships found with local grey matter in any of the AAL regions.


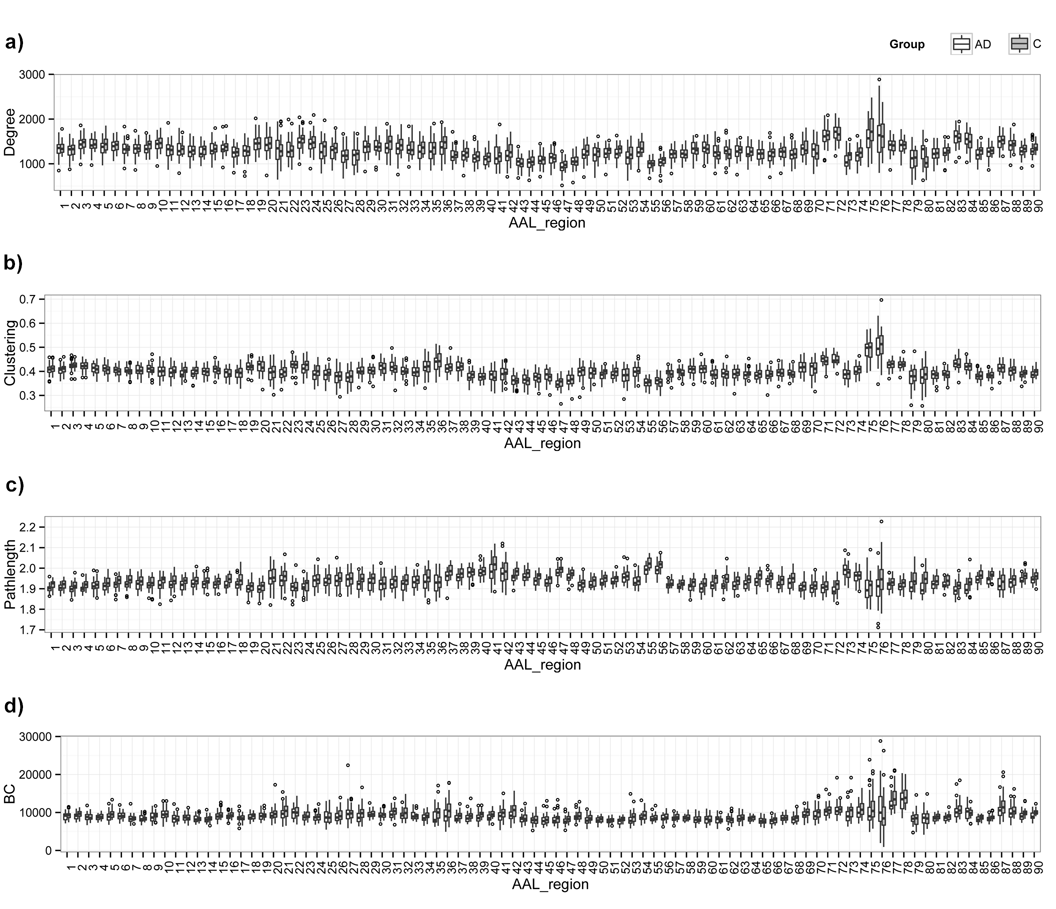


**Figure S2.** **Box plots to the distributions of local graph property values for all AAL regions.** Box plots are shown for the Alzheimer’s disease (AD; white boxes) and control (C; grey boxes) groups for the following graph properties: a) the degree, b) the clustering coefficient, c) the path length and d) the betweenness centrality (BC). For all AAL area labels the even numbers indicate the left and odd numbers indicate the right hemisphere. 1 & 2 = precentral gyrus, 3 & 4 = superior frontal gyrus, 5 & 6 = superior orbitofrontal gyrus, 7 & 8 = middle frontal gyrus, 9 & 10 = middle orbitofrontal gyrus 11 & 12 = inferior frontal operculum, 13 & 14 = inferior frontal triangularis, 15 & 16 = inferior orbitofrontal gyrus, 17 & 18 = Rolandic operculum, 19 & 20 = supplementary motor area, 21 & 22 = olfactory sulcus, 23 & 24 = medial superior frontal gyrus, 25 & 26 = medial orbitofrontal gyrus, 27 & 28 = gyrus rectus, 29 & 30 = insula, 31 & 32 = anterior cingulate, 33 & 34 = middle cingulate,

35 & 36 = posterior cingulate, 37 & 38 = hippocampus, 39 & 40 = parahippocampal gyrus, 41 & 42 = amygdala, 43 & 44 = calcarine sulcus, 45 & 46 = cuneus, 47 & 48 = lingual gyrus, 49 & 50 = superior occipital gyrus, 51 & 52 = middle occipital gyrus, 53 & 54 = inferior occipital gyrus, 55 & 56 = fusiform gyrus, 57 & 58 = postcentral gyrus, 59 & 60 = superior parietal gyrus, 61 & 62 = inferior parietal gyrus, 63 & 64 = supramarginal gyrus, 65 & 66 = angular gyrus, 67 & 68 = precuneus, 69 & 70 = paracentral lobule, 71 & 72 = caudate, 73 & 74 = putamen, 75 & 76 = pallidum, 77 & 78 = thalamus, 79 & 80 = Heschl’s gyrus, 81 & 82 = superior temporal gyrus, 83 & 84 = superior temporal pole, 85 & 86 = middle temporal gyrus, 87 & 88 = middle temporal pole,

89 & 90 = inferior temporal gyrus.
